# Editorial: Immune Mechanisms in the Pathologic Response to Particles, Fibers, and Nanomaterials

**DOI:** 10.3389/fimmu.2021.665810

**Published:** 2021-03-19

**Authors:** Qiang Ma, Kenneth Michael Pollard, Jared M. Brown, Paola Italiani, Seyed Moein Moghimi

**Affiliations:** ^1^Health Effects Laboratory Division, National Institute for Occupational Safety and Health, Morgantown, WV, United States; ^2^Department of Molecular Medicine, The Scripps Research Institute, La Jolla, CA, United States; ^3^Skaggs School of Pharmacy, University of Colorado Anschutz Medical Campus, Aurora, CO, United States; ^4^Institute of Biochemistry and Cell Biology, National Research Council (CNR), Naples, Italy; ^5^School of Pharmacy, Newcastle University, Newcastle upon Tyne, United Kingdom

**Keywords:** particle, nanoparticle, nanomedicine, immune response, autoimmunity, allergy, inflammation, particle immunotoxicity

It may come as a surprise that a Research Topic in Frontiers in Immunology is devoted to “Immune mechanisms” in the pathologic response to “particles, fibers, and nanomaterials,” a large group of natural and man-made particulates with dimensions in micro and nano ranges. Unlike pathogens, many particulate materials do not possess apparent antigenic structures and are assumed to be non-immunogenic. But because of their small size and airborne propensity, inhaled particulates can penetrate deep into the lung and pleura to cause inflammation, fibrosis, and cancer, exemplified by silicosis, asbestosis, and mesothelioma that are progressive and have high mortality rates (1, 2). Notwithstanding, the role of immune responses in the pathogenesis of particulate-induced diseases has gained increasing attention. A plethora of studies have shown that particulates activate various immune cells to promote physiological responses to particulate deposition and, under pathological conditions, development of fibrosis, malignancy, and autoimmunity (3, 4). The past two decades have also witnessed rapid advancement of nanotechnology and commercialization of nanomaterials, raising concerns on their possible adverse immune effects in exposed individuals (5–7). Understanding the interactions between particulates and immune functions has emerged as a new frontier for researchers in immunology, toxicology, and nanoscience. This Research Topic discusses recent trends in particle immunotoxicology with focus on pathologic immune effects of particulates and mechanisms mediating particle-immune interactions.

## Immune Effects and Disease Models

Exposure to silica and asbestos is associated with increased incidence of autoimmune abnormalities (3, 4, 8, 9). We begin with a perspective article by Pollard highlighting the current state of knowledge regarding the effects of selected particle, fiber, and nanomaterial exposures on the immune system, specifically autoimmunity. Pathological processes following exposure to particulate and fibrous materials, known to be associated with pre-clinical autoimmunity and autoimmune diseases, were contrasted with those linked to engineered nanoparticles, where evidence for induction of autoimmunity is less convincing. In a research article, Rajasinghe et al. extended their previous observations that dietary docosahexaenoic acid (DHA), a ω-3 polyunsaturated fatty acid, can markedly ameliorate the exacerbated pulmonary, systemic, and renal manifestations of systemic autoimmune disease following crystalline silica exposure. Their findings indicate that DHA, at physiologically relevant concentrations, is capable of attenuating macrophage death, allowing phagocytosis of dead and dying cells and thereby reducing accumulation of cellular material that might stimulate autoreactivity.

Pseudoallergic and anaphylactoid immune responses to engineered nanomaterials have received considerable attention as severe allergy-like immune responses to medical formulation of nanomaterials have been reported. For example, Feraheme®, an iron oxide nanoparticle formulation used in patients with severe anemia, caused anaphylactoid reactions and death in a subset of patients following the first use (10). In this topic, Alsaleh and Brown discussed Type 1 allergic immune responses to nano exposure through mast cell degranulation and activation, highlighting a novel non-IgE-driven mechanism induced by nanoparticles. This suggests that a first-time exposure to nanoparticles to sensitize an individual is not required, which may explain some anaphylactoid responses observed in patients treated with Feraheme®. Joubert et al. reviewed the exposure, clinical, and mechanistic aspects of particle-associated allergic and pseudoallergic responses and disease. Mechanisms of sensitization and effector functions were discussed, underscoring Th2 allergic immune responses and key cells involved. The utility of particulates in anti-allergic therapy and immunity was presented.

Air pollution can increase acute asthmatic episodes and worsen asthmatic symptoms. Sachdeva et al. reviewed how basic cellular processes, such as autophagy, mitophagy, and cellular senescence, may play a crucial role in airway inflammation, airway hyper-responsiveness, and airway remodeling. Specifically, the authors discussed how co-exposure of environmental pollutants including particles and allergens may affect these responses in the development of asthmatic phenotypes and for therapeutic targeting against asthma.

Metabolic syndrome is a group of pathologic conditions characterized by high-blood pressure, dyslipidemia, and high blood glucose levels (11). Patients with metabolic syndrome are at increased risk of type-2 diabetes and cardiovascular disease. Thus, Alqahtani et al. examined the effect of metabolic syndrome-associated dyslipidemia on silver nanoparticle-induced immune responses. They conclude that metabolic syndrome exacerbates the acute toxicity of silver nanoparticle exposure through disruption of lipid mediators of inflammatory resolution, which leads to enhanced pulmonary inflammation.

Endogenous metabolites, such as monosodium urate (MSU) and cholesterol, can form crystals in tissue to cause diseases like gout and atherosclerosis. Shin et al. studied neutrophil infiltration in MSU-induced inflammatory gouty lesions in BALB/c mice. MSU acted as a damage-associated molecular pattern signal to activate the P2Y6 purinergic receptor and induce production of CXCL8, which stimulates neutrophil influx to the synovial fluid. Moreover, 1-palmitoyl-2-linoleoyl-3-acetyl-rac-glycerol (PLAG) suppressed MSU-induced acute gouty inflammation in mice by targeting P2Y6-mediated expression of CXCL8 and induction of neutrophil flux, providing a possible anti-gouty therapy.

Chronic obstructive pulmonary disease (COPD) is a major public health concern worldwide and may become the third leading cause of death by 2030 (12). COPD is triggered by repeated airway exposures to harmful particles, mainly from cigarette smoke. B-cell activating factor (BAFF), a cytokine involved in the maturation and survival of B lymphocytes, participates in the regulation of innate immune responses at the respiratory tract mucosa. Nascimento et al. investigated the role of BAFF in the pathophysiology of pulmonary disease in mice exposed to cigarette smoke. They observed that acute cigarette smoke exposure promotes BAFF expression in airway-recruited neutrophils *in vivo* and *in vitro*. Moreover, acute neutrophilic airway inflammation induced by cigarette smoke required functional BAFF.

McDaniel et al. focused on immunotoxicity of Magneli phase titanium suboxide nanoparticles, which were discovered recently in large quantities during burning of coal. Their findings reveal that macrophages were the most impacted cell type leading to oxidative stress, mitochondrial dysfunction and apoptosis *in vitro*, while *in vivo* studies using a chronic pulmonary exposure model in mice showed negative impacts on lung function resulting from oxidative stress and inflammatory responses.

## Mechanistic Aspects of Particle-Immune Interaction

The composite nature of many nanoparticles might allow non-specific adsorption of biomolecules, which could dramatically modulate immunological responses by nanoparticles (13). This notion has received much attention in nanomedicine, where adsorption of blood proteins to intravenously injected clinical and pre-clinical nanoparticles could not only direct and modulate complement activation through different pathways, but also phagocytic responses by different macrophage sub-populations (14). Depending on their surface properties, nanoparticles often acquire a complex coat of environmental biomolecules comprising opsonic and dysopsonic components in biological milieu. In this topic, Fadeel provided a brief overview on physicochemical and biological factors controlling immune recognition and immune evasion of nanoparticles, while Papini et al. critically discussed the modulatory roles of biomolecule corona on nanoparticle interaction with elements of innate immunity, and draw attention to species differences in blood opsonization events and macrophage recognition of opsonized nanoparticles.

Respirable particles may also adsorb or contract pathogens such as viral particles and thereby modulate the behaviors of pathogens. In this connection, Farhangrazi et al. proposed a hypothesis for the role of particulate matter pollutants in SARS-CoV-2 delivery to alveolar macrophages as an additional mechanism for spreading infection. They further considered particulate pollutants as possible antigen carriers and adjuvant for promoting immunity in the lungs.

The initial response to deposition of particulates in tissue is largely inflammatory in nature where immune cells coordinate the vascular, mucosal barrier, and cellular and molecular responses to help clear particulates and repair damaged tissue (15). Polarization of immune cells is key to the proper initiation, amplification, and resolution or progression of inflammation by enabling several programmed immune reactions (16). These include the Th1- and M1-mediated proinflammatory type 1 response, and Th2- and M2-mediated pro-resolving type 2 response. Exposure to particles also activates the polarization of a battery of other immune cells, such as Th17, Treg, Breg, and MDSC cells, to modulate the evolvement of inflammation and chronic lesions. A timely review by Ma encapsulates the progress of research in this direction, highlighting polarization of immune cells and how it modulates proinflammatory or pro-resolving responses and, thereby, controls the recovery or progression of fibrosis and cancer upon particle exposure.

Bioactive lipid molecules derived from polyunsaturated fatty acids (PUFAs) play critical roles in the regulation of inflammation (17). Lim et al. examined the initiation and resolution of inflammatory responses to multi-walled carbon nanotubes (MWCNTs) and fullerene C60 (C60F) in mouse lungs. The authors found that high toxicity MWCNTs at a low dose caused prolonged inflammatory lesions that progressed to granulomatous inflammation, whereas low toxicity C60F at high doses stimulates acute inflammation that largely resolves. Deposition of the nanoparticles in the lung stimulated production of proinflammatory lipid mediators during type 1 inflammation, but induced production of pro-resolving lipid mediators during type 2 inflammation, which were attributed to differential induction of 5- and 15-arachidonate lipoxygenase pathways in M1 or M2 macrophages, respectively. Thus, this study provides a molecular basis for regulation of lung inflammation by M1 and M2 macrophages *via* bioactive lipid mediators.

Nanoimmunosafety has become crucial in nanomedicine to avoid unwanted immune system activation. In this respect, Italiani et al. discussed the importance of evaluating the capacity of nanoparticles to induce or modulate the innate immune memory, i.e., the capacity of innate immune cells previously exposed to various stimuli to mount stronger and more effective responses (“potentiation”) or weaker and less self-damaging reactions (“tolerance”) upon second-time exposures. Starting from the assumptions that the innate memory is the result of metabolic and epigenetic reprogramming of innate immune cells, and that nanoparticles can affect metabolic and epigenetic changes, the authors report on effects of nanoparticles on induction and modulation of innate memory in murine and human macrophages.

The suitability of animal models in nanotoxicity and nanosafety evaluation has been a subject of debate because of apparent differences in structure and physiology between animal models and humans. Bedőcs and Szebeni discussed whether a pig model can be used as an appropriate animal model in nanomedicine safety assessment by examining an ill-fated clinical development of hemoglobin-based oxygen carriers (HBOCs). The authors draw an analogy between HBOC's hemodynamic effects aggravating hemorrhagic shock in trauma patients and nanomedicine-induced porcine complement (C) activation-related pseudoallergy leading to anaphylactoid shock in a pig model. The pros and cons of using such models to predict nanomedicine-induced hypersensitivity reactions were discussed.

In aggregate, the Research Topic summarized recent developments in understanding the immunologic effects of micro and nano particulates and discussed the mechanistic aspects of these effects. In view of the growing awareness of particle health effects, this topic provides new pathways and targets for safety evaluation, biomarkers, and pharmacotherapy for human diseases resulting from exposure to particles and nanoparticles. Given the vast numbers and variations of particulates and their immune effects, it is unlikely that this Research Topic has covered all aspects of the immune effects of particulates. Therefore, it is the hope of the authors and editors that this Research Topic serves as a starting point for particle immunotoxicology that will evolve rapidly in the coming years.

## Author Contributions

All authors listed have made a substantial, direct and intellectual contribution to the work, and approved it for publication.

## Conflict of Interest

The authors declare that the research was conducted in the absence of any commercial or financial relationships that could be construed as a potential conflict of interest.
